# UBE2L6/UBCH8 and ISG15 attenuate autophagy in esophageal cancer cells

**DOI:** 10.18632/oncotarget.15182

**Published:** 2017-02-08

**Authors:** Chloe M. Falvey, Tracey R. O'Donovan, Shereen El-Mashed, Michelle J. Nyhan, Seamus O'Reilly, Sharon L. McKenna

**Affiliations:** ^1^ Cork Cancer Research Centre, University College Cork, Cork, Ireland; ^2^ Department of Oncology, Cork University Hospital, Cork, Ireland

**Keywords:** esophageal, autophagy, apoptosis, ISG15, UBE2L6

## Abstract

Esophageal cancer remains a poor prognosis cancer due to advanced stage of presentation and drug resistant disease. To understand the molecular mechanisms influencing response to chemotherapy, we examined genes that are differentially expressed between drug sensitive, apoptosis competent esophageal cancer cells (OE21, OE33, FLO-1) and those which are more resistant and do not exhibit apoptosis (KYSE450 and OE19). Members of the ISG15 (ubiquitin-like) protein modification pathway, including UBE2L6 and ISG15, were found to be more highly expressed in the drug sensitive cell lines. In this study, we evaluated the contribution of these proteins to the response of drug sensitive cells. Depletion of UBE2L6 or ISG15 with siRNA did not influence caspase-3 activation or nuclear fragmentation following treatment with 5-fluorouracil (5-FU). We assessed autophagy by analysis of LC3II expression and Cyto-ID staining. Depletion of either ISG15 or UBE2L6 resulted in enhanced endogenous autophagic flux. An increase in autophagic flux was also observed following treatment with cytotoxic drugs (5-FU, rapamycin). In ISG15 depleted cells, this increase in autophagy was associated with improved recovery of drug treated cells. In contrast, UBE2L6 depleted cells, did not show enhanced recovery. UBE2L6 may therefore influence additional targets that limit the pro-survival effect of ISG15 depletion. These data identify UBE2L6 and ISG15 as novel inhibitors of autophagy, with the potential to influence chemosensitivity in esophageal cancer cells.

## INTRODUCTION

Cancers of the esophagogastric region are highly malignant tumours with European 1-year and 5-year survival rates of 40 % and 12 % respectively, according to the 2015 EUROCARE-5 study [1]. The current gold standard of treatment involves both chemotherapeutic intervention and surgery to remove the primary tumour [2, 3]. However, in the long term, this approach is often unsuccessful due to the survival of drug resistant disseminated cells that can recover from treatment [4]. There is therefore a major need to develop new approaches to treat both primary and recurrent esophageal cancer.

A highly significant factor contributing to drug resistance is the inactivation of apoptosis in cancer cells. We have previously shown that 5-fluorouracil and cisplatin induce apoptotic cell death in drug sensitive OE21 and OE33 esophageal cancer cell lines. In contrast, treatment of apoptosis resistant cell lines KYSE450 and OE19 results in induction of autophagy and subsequent recovery following withdrawal of the drug [5]. Autophagy is a highly conserved process that catabolises and recycles damaged organelles and protein complexes. This process can be triggered when a cell is exposed to stressful conditions such as nutrient deprivation, cellular damage and hypoxia [6]. In the case of a healthy cell, this can have a positive, protective function. However, an established cancer cell can exploit this process to facilitate survival when treated with a cytotoxic agent, resulting in tumour cell resistance and cancer recurrence [7, 8].

We undertook gene expression analysis of the sensitive and resistant esophageal cancer cells lines in order to identify patterns of gene expression associated with cellular response to cytotoxic drug. We found that several members of the ISG15 conjugation pathway are upregulated in apoptosis & autophagy competent cell lines (OE21 and OE33) and downregulated in cell lines which are apoptosis resistant and which respond to drug treatment by inducing autophagy only (KYSE450 and OE19).

Interferon Stimulated Gene 15 (ISG15) is a 15 kD ubiquitin-like protein modifier which can be conjugated to protein substrates in order to modify their functions. Stimulation by Type I Interferons (IFN α or β) activates the transcription of over 2000 Interferon Stimulated Genes (ISGs) (reviewed in [9, 10, 11]). Among these ISGs are three key enzymes: UBE1L (ISG15 specific E1 activating enzyme), UBE2L6 (ISG15/ubiquitin E2 conjugating enzyme) and HERC5 (ISG15 specific E3 ligase). In a mechanism similar to ubiquitination, ISG15 is conjugated to targets via the sequential co-operation of these E1, E2 and E3 enzymes. Finally, USP18 (an ISG15 specific isopeptidase enzyme) can reverse ISG15 conjugation of target proteins.

Over 300 substrates for ISG15 modification have been reported. These include existing and newly synthesised proteins involved in proliferation, chromatin remodelling, cell cycle regulation and innate immune system activation [12, 13, 14]. The ISG15 pathway has been found only in higher eukaryotic cells, indicating specialised functions [15]. Importantly, UBE2L6 is not restricted to ISGylation as it can also function as an E2 enzyme in the conjugation of ubiquitin to target proteins [16].

In this study, we have identified ISG15 and UBE2L6 as negative regulators of autophagy in esophageal cancer cells. As the effects of UBE2L6 siRNA on autophagy are consistent with ISG15 siRNA, it is possible that UBE2L6 influences autophagy by promoting direct conjugation of ISG15 to targets which directly or indirectly regulate autophagy.

It is notable however, that the effects of ISG15 and UBE2L6 depletion on overall survival are different. This is perhaps unsurprising as UBE2L6 can facilitate ubiquitination, which will expand its repertoire of targets and thus impact its overall biological effect.

## RESULTS

### Proteins associated with the ISG15 conjugation pathway are upregulated in apoptosis competent cell lines

**Figure 1:**
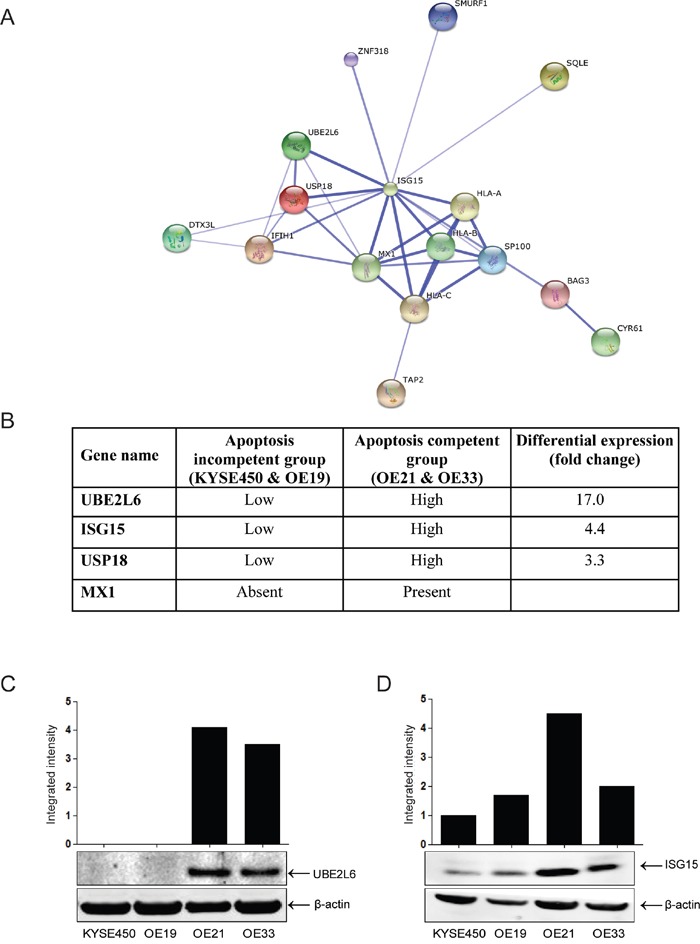
Proteins associated with the ISG15 conjugation pathway are upregulated in apoptosis competent cell lines **A**. String analysis of differentially expressed proteins that intersect with ISG15 (http://string-db.org). This is the confidence view. Stronger associations are represented by thicker lines. **B**. Genes which show at least a 3-fold differential in expression and that are involved in the ISG15 conjugation network are illustrated in the table. **C**. Differential expression of UBE2L6 and **D**. ISG15 were confirmed by Western blot. Bands were quantified and normalised to β-actin.

We previously evaluated the response of four esophageal cell lines to drug treatment [5]. Two cell lines (OE21 and OE33) exhibited both apoptosis and autophagy and are relatively drug sensitive. In contrast, two cell lines (KYSE450 and OE19) fail to undergo apoptosis in response to drug treatment and instead, respond by inducing autophagy alone. These cell lines are more drug resistant and can recover following drug withdrawal. We reasoned that these two groups may differ in the genes that they constitutively express, and this may affect their response to cytotoxic stress. We conducted Affymetrix gene array analysis of all four cell lines in triplicate. The array hybridisation and bioinformatics was carried out by Almac Diagnostics (www.almac.com; methods are included in Supplementary Figure 1). We initially identified a list of genes that were common to group A (apoptosis & autophagy cells) and a separate list of genes that were common to group B (autophagy only cells). Each group contained one adenocarcinoma and one squamous cell carcinoma cell line. Genes which differed due to histological subtype were eliminated at this stage. We then looked for genes that are always expressed in the same direction in Group A (apoptosis & autophagy competent) but differentially expressed in Group B. 260 genes had more than a three-fold difference in the level of expression between the apoptosis competent (OE21, OE33) and apoptosis incompetent, autophagy only (KYSE450 and OE19) cell lines. Biological data on these genes was assimilated and STRING analysis was used to look for associated protein networks. One of the nodes identified by STRING included the most differentially expressed gene (UBE2L6; 17-fold differential) and several other members of the ISGylation network including ISG15 itself (Figures 1A and 1B). We therefore selected UBE2L6 and ISG15 for further analysis. Differential expression in the cell lines was confirmed by Western blot. These data confirm a significant difference in UBE2L6 and ISG15 protein expression between the apoptosis competent and apoptosis incompetent cell lines (Figures 1C and 1D).

### siRNA targeted against UBE2L6 or ISG15 has no influence on the induction of apoptosis

**Figure 2:**
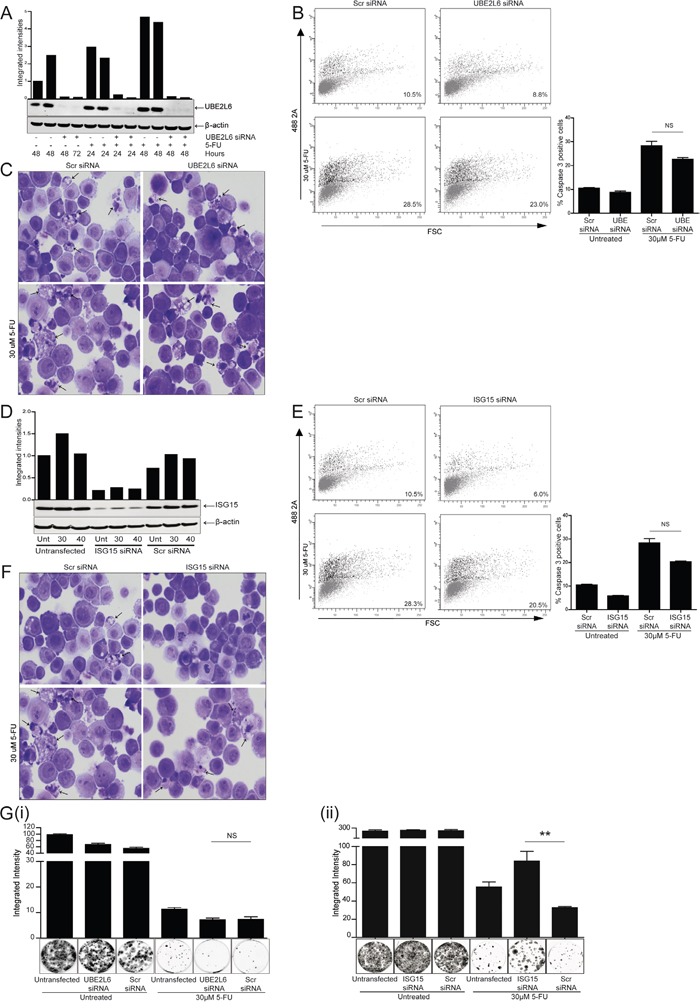
siRNA depletion of UBE2L6 or ISG15 has no effect on the induction of apoptosis in OE21 cells treated with 5-fluorouracil (5-FU) **A**. Gene specific siRNA (20 nM), was used to deplete UBE2L6 expression. Silencing was confirmed by Western blot in the presence and absence of 5-FU. **B**. UBE2L6 depleted and scramble control cells were treated with 5-FU (30 μM) for 48 hr and were analysed for levels of active caspase-3 by flow cytometry. Data from three independent experiments is presented as ‘% caspase positive cells’. **C**. Morphological analysis (40x magnification) of scramble control siRNA (left panels) and UBE2L6 siRNA (right panels) cells in the absence (upper panels) or presence of 5-FU (lower panels) (arrows indicate nuclear fragmentation). **D**. siRNA was used to deplete ISG15 expression and silencing was confirmed by Western blot in the presence and absence of 5-FU. All bands were quantified and normalised to β-actin. **E**. ISG15 depleted and scramble control cells were treated with 5-FU for 48 hr and were analysed for levels of active caspase-3 by flow cytometry. Data from three independent experiments is presented as ‘% caspase positive cells’. **F**. Morphological analysis (40x magnification) of scramble control siRNA (left panels) and ISG15 siRNA (right panels) cells in the absence (upper panels) and presence of 5-FU (lower panels) (arrows indicate nuclear fragmentation). **G**. Following drug removal, a colony formation assay was carried out to determine the ability of UBE2L6 depleted (i) or ISG15 depleted (ii) cells to recover, relative to scramble control cells. Colonies were stained using Rapi-diff and quantified using the Odyssey Infra-red imaging system. Triplicate data is presented in the graph as integrated intensity ± SEM (**p < 0.01).

We initially examined whether the expression levels of UBE2L6 or ISG15 were important for the induction of apoptosis in OE21 cells treated with 5-fluorouracil (5-FU). Activation of caspase-3, a standard biochemical marker of apoptosis, was quantitated by flow cytometry and morphological features of cell death examined, in drug treated cells, in the presence or absence of UBE2L6 or ISG15 siRNA (Figure 2).

siRNA mediated depletion of UBE2L6 was confirmed by Western blot (Figure 2A). OE21 cells are relatively drug sensitive and activate caspase-3 in response to treatment with 5-FU. OE21 cells with UBE2L6 siRNA alone or in the presence of 5-FU (30 μM) for 48 hr (upper and lower right panels), induced similar levels of activated caspase-3 as the scrambled controls (upper and lower left panels). Data from triplicate experiments is presented as “% caspase positive cells” to the right. Examination of cell morphology also identified similar levels of apoptotic nuclear fragmentation in UBE2L6 depleted and scrambled control cells treated with 5-FU (Figure 2C lower right and left panels respectively, arrows).

Activation of caspase-3 was also examined in ISG15 depleted cells. siRNA mediated depletion of ISG15 was confirmed by Western blot (Figure 2D). ISG15 depleted cells showed similar levels of active caspase-3 to scrambled control cells in the absence (Figure 2E upper panels) or presence of 5-FU (30 μM) (Figure 2E lower panels). Data from triplicate experiments is presented to the right as “% caspase-3 positive cells”. Examination of cell morphology also identified similar levels of apoptosis in ISG15 depleted and scrambled control cells treated with 5-FU (Figure 2F arrows).

A colony formation assay was performed to evaluate the ability of these cells to recover from treatment with 5-FU. In cells transfected with UBE2L6 siRNA, there was no significant difference in the recovery of cells following 5-FU treatment [Figure 2G (i)]. In contrast, cells transfected with ISG15 siRNA demonstrated a significant rescue from 5-FU relative to scrambled controls (**p = 0.0089) [Figure 2G (ii)] (data is presented as integrated intensity and colonies shown are representative of three replicates).

### ISG15 silencing with siRNA promotes endogenous and induced autophagic flux

**Figure 3:**
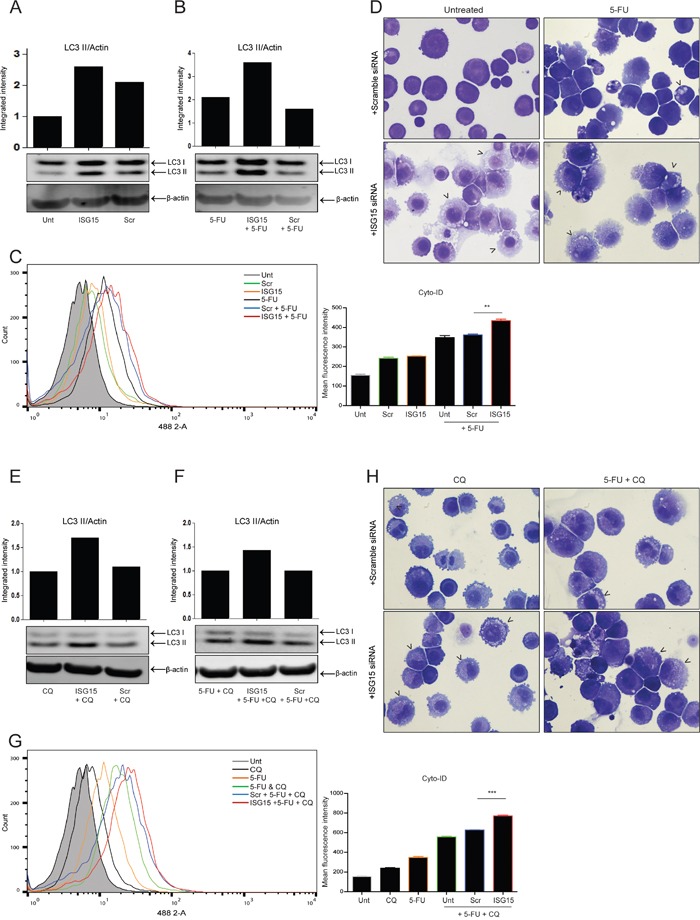
ISG15 silencing with siRNA promotes both endogenous and induced autophagic flux For all autophagy assays - OE21 cells were treated for 24 hr and allowed to recover for an additional 24 hr in drug free medium. The effect of ISG15 siRNA (middle lanes) on **A**. endogenous LC3 II and **B**. 5-FU-induced (30 μM) LC3 II, relative to untransfected (left lanes) and scrambled control (right lanes) was assessed by Western blot. **C**. Cyto-ID autophagy detection kit was used to assess autophagosome formation by ISG15 siRNA alone (orange overlay) relative to scrambled (green overlay) or untreated cells (grey filled histogram). 5-FU-induced autophagy was assessed in ISG15 depleted cells (red overlay) relative to scramble control (blue overlay) (**p < 0.005) or untransfected (black overlay) cells. **D**. Analysis of vesicle accumulation (black arrowheads) in scrambled (upper panels) and ISG15 siRNA (lower panels) cells, without (left panels) and with 5-FU (right panels) (40x magnification). **E**. Autophagic flux was assessed by measuring LC3 II levels in untransfected (left lanes), scrambled control (right lanes) and ISG15 siRNA (middle lanes) cells following 24 hr treatment with chloroquine (10 μM) alone or **F**. in combination with 5-FU (30 μM). LC3 II levels were normalised to β-actin and presented as integrated intensities. Western blots shown in parts A, B, E & F are all from the same blot **G**. Cyto-ID assay was used to assess 5-FU-induced autophagic flux in ISG15 depleted (red overlay), scramble control (blue overlay) (***p < 0.0001) and untransfected (green overlay) cells, treated with 5-FU and chloroquine. **H**. Induction of vesicles was confirmed by morphological analysis (black arrowheads, 40x magnification) following transfection with scrambled control (upper panels) or ISG15 siRNA (lower panels) in cells treated with chloroquine alone (left panels) or in combination with 5-FU (right panels).

The enhanced recovery of cells with ISG15 siRNA suggested that ISG15 expression may be impeding a survival pathway. Therefore, the effect of ISG15 siRNA on endogenous and induced autophagy was assessed. For all autophagy assays, cells were treated with 5-FU (30 μM) for 24 hr and then replenished with drug free media for a further 24 hr. As shown in Figure 3A, analysis of LC3 II (an autophagosome marker) by Western blot revealed a clear increase in LC3 II accumulation in untreated cells with ISG15 siRNA alone when compared with untransfected and scrambled controls. LC3 II induction by 5-FU was also further enhanced by ISG15 siRNA (Figure 3B middle lane).

The effect of ISG15 siRNA on autophagy was also quantified using an additional assay, the Cyto-ID autophagy detection kit, which specifically labels autophagosomes. As shown in Figure 3C, ISG15 siRNA significantly increased 5-FU induced Cyto-ID fluorescence confirming enhanced autophagosome accumulation (red overlay) beyond scramble control (blue overlay) (**p = 0.0016). Data from three independent experiments is presented as mean fluorescence intensity (MFI) to the right.

Analysis of cellular morphology showed vesicle accumulation in cells treated with 5-FU (Figure 3D upper right panel, arrowheads). When ISG15 is depleted by siRNA, vesicle accumulation is further enhanced (Figure 3D lower left and right panels, arrowheads).

The entire autophagic process, from sequestration to degradation is referred to as autophagic flux. LC3 II accumulation may be a consequence of either increased autophagy initiation or a block in autophagosome turnover. Therefore, to differentiate between induction and vesicle accumulation due to a failure in turnover, cells were pre-treated with chloroquine to block lysosome function and autophagosome turnover. Any LC3 II accumulation beyond that observed with chloroquine alone is then attributed to enhanced autophagy initiation [17].

The effect of ISG15 siRNA on flux was assessed by 2 hr pre-treatment of cells with chloroquine (10 μM) followed by treatment with 5-FU for 24hr, which was followed by an additional 24 hr recovery in drug free medium. As shown in Figure 3E, treatment of untransfected or scrambled control cells with chloroquine alone showed LC3 II accumulation (when compared to control levels in Figure 3A), which was further enhanced by ISG15 siRNA (lane 2). In cells treated with 5-FU in the presence of chloroquine (Figure 3F), LC3II levels were also further enhanced by ISG15 siRNA, indicating elevated autophagic flux.

The Cyto-ID assay was used to confirm these findings (Figure 3G). A significant increase in fluorescence is observed in ISG15 depleted cells treated with a combination of 5-FU and chloroquine (red overlay) when compared to scramble controls (blue overlay) (***p < 0.0001). Data from three independent experiments is presented as mean fluorescence intensity (MFI) to the right.

Analysis of cell morphology indicated extensive accumulation of vesicles in the presence of both chloroquine and a combination of 5-FU & chloroquine in ISG15 depleted cells (Figure 3H lower left and right panels, arrowheads). Collectively, these data suggest that ISG15 siRNA is initiating autophagy rather than impeding autophagosome turnover. Similar effects of ISG15 siRNA on induced autophagy were observed with rapamycin and valproic acid (Supplementary Figure 2).

An additional apoptosis competent esophageal cancer cell line (FLO-1), expressing the ISGylation pathway was also evaluated. Depletion of ISG15 was confirmed by Western blot (Supplementary Figure 3A) and resulted in elevated LC3 II in both untreated and 5-FU treated cells, when compared to scramble controls (Supplementary Figure 3B). The Cyto-ID assay was used to confirm that ISG15 siRNA is also initiating autophagy in this cell line, with enhanced fluorescence observed in ISG15 depleted cells treated with either chloroquine alone [Supplementary Figure 3C (i)] (red overlay) (**p = 0.0046) or combined with 5-FU [Supplementary Figure 3C (ii)] (red overlay) (**p = 0.0038) relative to scrambled controls (blue overlays). Depletion of ISG15 also enhanced the recovery of FLO-1 cells, relative to scrambled controls (Supplementary Figure 3D).

### UBE2L6 siRNA promotes endogenous autophagy induction

Our data thus far has suggested that ISG15 can influence autophagy. If UBE2L6 is required for conjugating ISG15 to substrate proteins, then UBE2L6 would be expected to influence autophagy in a similar manner to ISG15.

**Figure 4:**
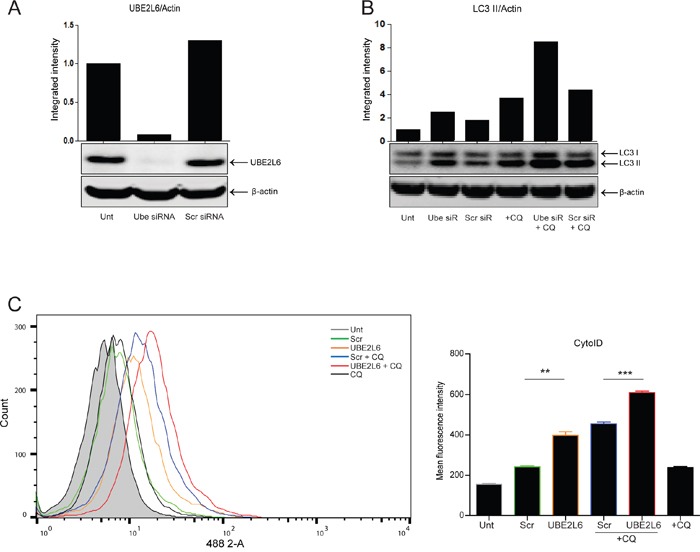
UBE2L6 siRNA promotes endogenous autophagy induction **A**. Silencing of UBE2L6 by siRNA (20 nM) was confirmed by Western blot. **B**. Induction of autophagy was evaluated by Western blot analysis of LC3 II levels normalised to β-actin for untreated (lanes 1-3) or 24 hr chloroquine (10 μM) treated cells (lanes 4-6) in the presence of UBE2L6 siRNA (lanes 2 & 5). **C**. Cyto-ID was used to assess autophagosome formation by UBE2L6 siRNA alone (orange overlay) relative to scrambled (green overlay) or untreated (grey filled peak). Flux was analysed in UBE2L6 siRNA cells (red overlay) and compared with scrambled siRNA control (blue overlay). Data from three independent experiments is presented to the right as mean fluorescence intensities ± SEM (**p < 0.005, ***p < 0.0005).

To evaluate the contribution of UBE2L6 to autophagy, gene expression was depleted by siRNA and loss of UBE2L6 protein was confirmed by Western blot (Figure 4A). UBE2L6 siRNA alone [Figure 4B lane 2 (without drug)] increased the level of LC3 II relative to controls (lanes 1 and 3). Chloroquine treatment (10 μM) alone increased LC3 II in scrambled and untransfected control cells (lanes 4 and 6) and this is significantly enhanced by UBE2L6 siRNA (lane 5), suggesting that UBE2L6 also influences autophagy initiation rather than turnover.

The effect of UBE2L6 siRNA on autophagy was also quantitated using the Cyto-ID assay. As shown in Figure 4C, UBE2L6 siRNA significantly increased Cyto-ID fluorescence (orange overlay) relative to scrambled control (green overlay) (**p = 0.0012). Treatment of UBE2L6 depleted cells with chloroquine achieved a further increase (red overlay) relative to the scrambled control (blue overlay) (***p = 0.0002). Data from three independent experiments is presented as mean fluorescence intensity (MFI) to the right. Collectively, these data indicate that UBE2L6 siRNA can promote endogenous autophagy induction, as observed with ISG15.

### UBE2L6 siRNA enhances autophagy induction by 5-FU

**Figure 5:**
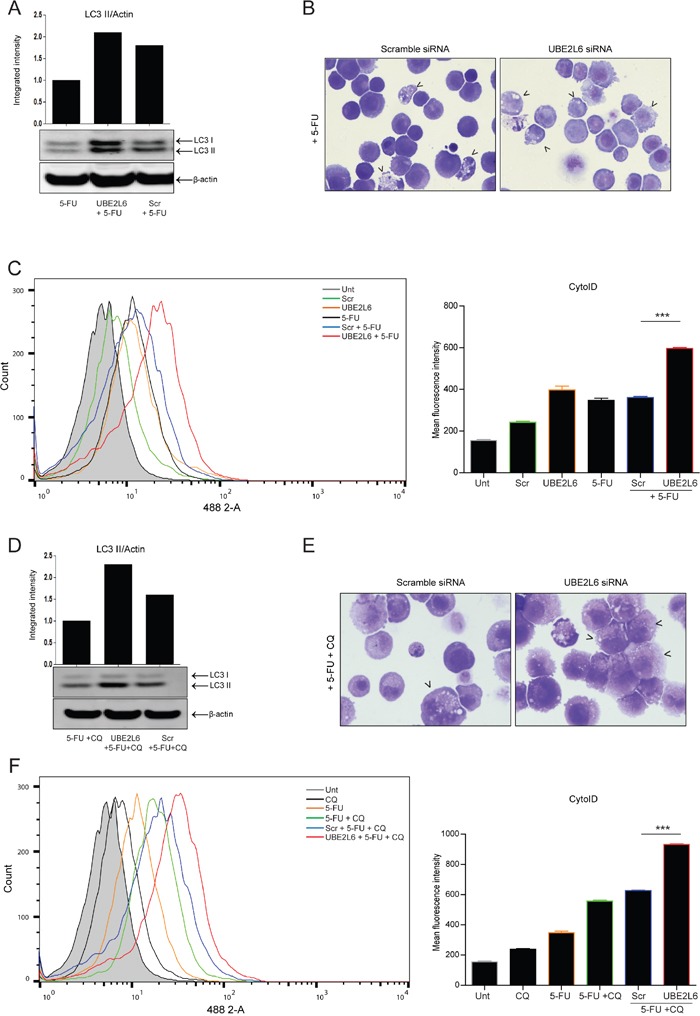
UBE2L6 siRNA enhances 5-FU-induced autophagic flux **A**. The effect of UBE2L6 siRNA (middle lane) on autophagy induction relative to untransfected or scrambled controls (left and right lanes respectively) following treatment with 5-FU (20 μM) for 24 hr was assessed by Western blot. **B**. Morphological analysis (40x magnification) compares vesicle accumulation (black arrowheads) in scrambled siRNA control cells (left panel) to UBE2L6 siRNA cells (right panel) following treatment with 5-FU. **C**. Cells treated with 5-FU (black overlay) were stained with Cyto-ID. Autophagosome formation was assessed in drug treated cells with UBE2L6 siRNA (red overlay), relative to scramble controls (blue overlay). Data from three independent experiments is presented as mean fluorescence intensity ± SEM to the right (***p < 0.0001). **D**. To assess flux, cells were treated with chloroquine (10 μM) in combination with 5-FU for 24 hr in the presence of either scrambled (lane 3) or UBE2L6 siRNA (lane 2). LC3 II levels were normalised to β-actin. Western blots shown in parts A & D are from the same blot **E**. Morphology of cells treated with a combination of 5-FU and chloroquine was compared in scrambled control cells (left panel) and UBE2L6 siRNA cells (right panel). Autophagic vesicles are indicated by black arrowheads (40x magnification). **F**. The effect of UBE2L6 siRNA on autophagic flux was assessed by treating cells with either 5-FU (orange overlay) or chloroquine (black overlay) alone or a combination of both (green overlay) in the absence (blue overlay) or presence (red overlay) of UBE2L6 siRNA. Mean Fluorescence Intensities of three independent experiments are shown to the right (***p < 0.0001).

We then assessed if UBE2L6 siRNA could enhance autophagy induction by a chemotherapeutic agent. OE21 cells were treated with 5-FU (20 μM) in the presence or absence of chloroquine (10 μM) for 24hr. As shown by Western blot in Figure 5A, UBE2L6 siRNA further enhanced LC3 II induction by 5-FU, indicating that UBE2L6 siRNA increases 5-FU induced autophagy, similar to ISG15. Examination of morphology of 5-FU treated cells showed enhanced vesicle formation in the presence of UBE2L6 siRNA (Figure 5B right panel, arrowheads).

Cyto-ID was also used to quantitate autophagosome formation induced by 5-FU (Figure 5C). In the presence of UBE2L6 siRNA (red overlay), 5-FU-induced autophagosome levels were greater than either 5-FU alone (black overlay) (***p < 0.0001) or scrambled controls (blue overlay) (***p < 0.001). Data from three independent experiments is presented as MFI to the right. Similar results were obtained for other autophagy inducers including rapamycin (Supplementary Figure 4), lithium chloride and valproic acid (not shown).

Effects on autophagic flux were assessed by Western blot. The combination of chloroquine and 5-FU, in untransfected or scrambled control cells, induced an increase in LC3 II above that observed with 5-FU alone (see Figure 5A), however the levels of LC3 II were further increased in UBE2L6 depleted cells (Figure 5D). Morphology also confirmed a further induction of vesicles in UBE2L6 depleted cells, when compared to scrambled control cells treated with a combination of chloroquine and 5-FU (Figure 5E right panel, arrowheads). Flux was also assessed using Cyto-ID staining. Chloroquine (black overlay) or 5-FU alone (orange overlay) caused an increase in fluorescence above untreated cells (grey filled peak) (Figure 5F). The combination of chloroquine and 5-FU (green overlay) resulted in autophagosome accumulation in excess of either chloroquine or drug alone, which was further enhanced by UBE2L6 siRNA (red overlay) relative to scrambled controls (blue overlay) (***p < 0.0001). Data from three independent experiments is presented as MFI to the right. The effect of UBE2L6 siRNA on autophagy induction was also confirmed using rapamycin (100 nM) in the presence or absence of chloroquine (10 μM) (Supplementary Figure 4).

The effect of UBE2L6 was also evaluated in the FLO-1 cell line. Silencing of UBE2L6 with siRNA was confirmed by Western blot (Supplementary Figure 5A). UBE2L6 siRNA alone increased the levels of LC3 II relative to controls (Supplementary Figure 5B, lanes 1-3). 5-FU treatment increased LC3 II in scrambled and untransfected cells (lanes 4 and 5) and this was enhanced by UBE2L6 siRNA (lane 6), suggesting that UBE2L6 influences 5-FU-induced autophagy in this cell line. The increase in LC3 II in UBE2L6 depleted cells treated with a combination of 5-FU and chloroquine (Supplementary Figure 5C, lane 6) suggests that UBE2L6 also influences initiation rather than turnover in the FLO-1 cells.

The Cyto-ID assay also demonstrates a significant difference in autophagosome accumulation in FLO-1 cells with depleted UBE2L6 (orange overlay) when compared to scrambled controls (green overlay) (Supplementary Figure 5D) (**p = 0.0013). This elevation of autophagy was also evident in response to 5-FU (Supplementary Figure 5E) (**p = 0.0075) and again in cells treated with a combination of 5-FU and chloroquine (Supplementary Figure 5F) (***p < 0.0001)—indicating elevated autophagic flux in both untreated and 5-FU treated FLO-1 cells (Supplementary Figure 5A-5F). Analysis of recovery of FLO-1 cells following 5-FU treatment, found no difference in UBE2L6 depleted cells relative to scrambled controls (Supplementary Figure 5G).

These data collectively suggest that UBE2L6 can negatively regulate the initiation of endogenous autophagy and autophagy induced by a range of other cytotoxic agents. This is consistent with the effect of ISG15 siRNA and suggests that UBE2L6 may regulate autophagy induction via the ISG15 pathway.

Induction of autophagy by ISG15 siRNA is associated with enhanced recovery in both OE21 and FLO-1 cells. In contrast, UBE2L6 depletion does not protect either cell line from 5-FU—despite clear induction of autophagy. UBE2L6 may therefore have other substrates or targets that limit any protective effects of the induced autophagy.

### Evaluation of mRNA expression in tumours

We examined publicly available databases to look for a potential biological significance of UBE2L6 or ISG15 transcript expression. The Cancer Genome Atlas (TCGA) had sequence data—but limited expression data for esophageal or gastric cancer. We then looked at a gastric cancer database that has been assembled recently from several sources and includes studies using the same arrays that we used in our initial analysis of the esophageal cell lines (HGU133) [18]. This combined database is publicly available at:

http://kmplot.com/analysis/index.php?p=service&cancer=gastric.

While this is not the same tissue type as our study, it is a similar type of cancer in terms of origin and treatment. Expression of UBE2L6 / UBCH8 (Affy ID201649_at) was analysed in 876 patients. Patients were split according to median expression and there was no restriction on subtype. Kaplan Meier overall survival (OS) analysis is presented in Supplementary Figure 6. Higher expression of UBE2L6 was associated with better overall survival (hazard ratio (HR) = 0.8, P= 0.02) (Supplementary Figure 6A). In our cell lines, this would be associated with apoptosis competency and repression of autophagy. We do not know if this is linked to outcome in patients. UBE2L6 expression can be up regulated by Type I interferons and may also be an indication of immune cell activation. ISG15 expression was also analysed and while a similar trend was apparent, this did not achieve significance (P=0.094) (Supplementary Figure 6B).

## DISCUSSION

In this study, we have identified ISG15 and UBE2L6 as negative regulators of autophagy in esophageal cancer cells. This suggests that ISG15 may be a key ubiquitin-like modifier of autophagy and that its conjugation to substrates is likely to involve the E2 activity of UBE2L6. Ubiquitin and other ubiquitin-like conjugation systems are a well-known feature of autophagy regulation and overall regulation of proteostasis. In contrast, modification of proteins with ISG15 is less well understood and has been largely associated with innate immune defence mechanisms. However, recent studies have highlighted a potential link to autophagy as two key regulators of autophagy or mitophagy (Beclin 1 and Parkin) have now been identified as targets of ISGylation.

### Autophagy targets

Type I IFN has been reported to induce ISGylation of Beclin 1 at several lysine residues [19]. This protects BECN1 from ubiquitination at Lys63 and inhibits PI3KC kinase activity. As this suppresses autophagy it would be consistent with our data, which imply a negative regulatory role for both UBE2L6 and ISG15 in autophagy. These observations by Xu *et al* were dependent on exogenous stimulation of the ISG15 pathway by Type I interferon. This treatment will upregulate many new proteins and any of these may be subject to ISGylation. In our study, the ISG15 pathway was investigated under conditions of endogenous upregulation. It is possible, however, that overlapping pathways and targets are involved in both models. Our study also provides the first evidence for involvement of UBE2L6 whereas the Xu *et al* study highlights the opposing role of USP18, as a positive regulator of autophagy. Together these studies support a key role for this pathway in autophagy regulation. Another study has reported that the E3 ligase Parkin, an important regulator of mitophagy, is also modified by ISGylation. Parkin was shown to be modified when ISGylation components were overexpressed or when cells are treated with type I IFN, LPS or other selected drugs. ISGylation at Lys-349 and Lys 369 was reported to enhance Parkins E3 ligase activity [20].

### UBE2L6, ISG15 and cancer

Aberrant expression of UBE2L6 or other members of the ISGylation system have been reported in various cancers [21]. Significant upregulation of UBE2L6 was reported in prostate cancer and esophageal squamous cell carcinoma when compared to corresponding non-malignant tissues [22, 23]. ISG15 expression was also associated with differentiation grade and metastasis in Hepatocellular carcinoma [24] and had prognostic value in esophageal squamous cell carcinoma patients, particularly those who consume alcohol [25]. In nasopharyngeal cancer, high ISG15 correlated with frequent local cancer recurrence and shorter overall survival [26]. In pancreatic cancer, ISG15 was secreted by tumour associated macrophages and promoted cancer stem cell renewal and invasiveness [27]. We interrogated a publically available database to see if we could identify a relationship between expression of UBE2L6 or ISG15 and overall survival in gastric cancer. Expression of UBE2L6 was significantly associated with better survival in this cohort. It would be useful to conduct further IHC analysis on patient tissue to establish whether this expression is primarily in the tumour tissue or also in stromal or immune infiltrating cells.

### ISGylation and therapeutic response

A previous study evaluated the effects of silencing either ISG15 or UBE2L6 on drug sensitivity in breast cancer cells. They reported a significant decrease in sensitivity to camptothecin (CPT) when either ISG15 or UBE2L6 were silenced. They also observed a reduction in the levels of ISG15 in a number of drug resistant cancer cells lines suggesting that ISG15 expression in tumours could be a factor affecting CPT sensitivity in these tumour cells [28]. In our study we found that while depletion of both IGS15 and UBE2L6 elevates autophagy, only those esophageal cancer cells with ISG15 knockdown showed a decrease in sensitivity to 5-FU, consistent with the effects of ISG15 silencing reported by Desai et al. In contrast, sensitivity of esophageal cells to 5-FU was unaffected by UBE2L6 knockdown. While we do not know the reason for this—it is clear from other studies that UBE2L6 can also act as an E2 enzyme for ubiquitin. The ubiquitination and stability of p21 has been reported to be mediated by UBE2L6 or UBCH7 and the E3 ubiquitin ligase p53RFP (p53-inducible RING-finger protein) [29]. The potential of UBE2L6 to influence ubiquitination of targets in a given cell type will therefore broaden its biological activity and may influence its overall effect on survival.

### Potential targets in cancer cells?

Interferons are pleiotropic cytokines that interfere with viral replication. They induce transcription of more than 2000 interferon stimulated genes which predominantly serve to activate the immune system. A number of groups have now identified Type I interferons as autophagy inducers [30]. In recent years, it has emerged that autophagy plays a crucial role in the clearance of bacterial and viral pathogens (reviewed in [31–33]). Many studies have also implicated UBE2L6 and ISG15 as critical components of the innate immune response to pathogen infection [34–36]. However, if expression of the ISG15 pathway was directly required for autophagy induction, we would have expected silencing of either UBE2L6 or ISG15 to inhibit autophagy in our cells rather than inducing it. Indeed, a recent study looking at enforced overexpression of ISG15, or Type I IFN stimulation, has reported that ISG15 interacts with HDAC6 and p62 which facilitates aggresome formation and thereby promotes autophagy [37]. As ISG15 has a preference for newly synthesised proteins [38], this may be a way of eliminating viral proteins which are produced in vast excess following infection, as they could potentially be cleared by ISGylation, aggregation and autophagy.

Here, with endogenous expression in esophageal cancer cells, we report the opposite effect; knockdown of ISG15 or UBE2L6 promotes autophagy. It is important to consider that interferon signalling results in elevated expression of a vast number of genes, any of which may be the primary inducers of autophagy during infection. It is possible that the ISG15 pathway can perform a negative regulatory role to prevent over activation of autophagy. This sort of negative feedback loop is a common feature in immune regulation. It is also possible that endogenous ISGylation (as in these tumour cells) may have a different function in tumour cells—and distinct targets—compared to interferon-induced ISGylation. It is notable that, many of the targets identified by mass spectrometry in IFN treated cells, were also constitutively expressed and involved in processes such as stress response, metabolism, chromatin remodelling or RNA processing [12].

We currently do not know the mechanism by which the ISGylation pathway may be regulating autophagy and it is possible that several targets are involved. Combined proteomic studies have identified at least 300 proteins that are targeted by ISGylation [12, 14, 39], although only a fraction have been experimentally validated as substrates, with known effects of ISGylation [40]. ISGylation may modulate stability, activity or interaction with other proteins. It has been suggested that ISGylation could increase the stability of substrates as it opposes poly-ubiquitination [41]. Silencing of UBE2L6 or ISG15 by siRNA may therefore lead to increased poly-ubiquitination and degradation of a negative regulator(s) of autophagy. Further studies must be carried out to identify the substrates and primary mechanisms involved in autophagy regulation.

In conclusion, this data has shown that the ISG15 pathway may play a role in chemosensitivity by regulating autophagy and survival. It is notable that this pathway is not critical to normal cells and is apparently only required under certain conditions, such as during an innate immune response. As such, it provides a relatively tumour specific targeting opportunity. Clearly, further molecular studies are required so that strategic targeting can be achieved and drug resistance eliminated in esophageal cancer.

## MATERIALS AND METHODS

### Cell culture and reagents

Established human esophageal cancer cell lines OE19, OE21 and OE33 were obtained from the European Collection of Cell Cultures. KYSE450 and FLO-1 cells were from DSMZ (Deutsche Sammlung von Mikroorganismen und Zellkulturen GmbH). OE19, OE21, OE33 and FLO-1 cell lines were maintained in RPMI 1640 medium (Sigma R8758) and KYSE450 cells were maintained in 50:50 RPMI 1640:F-12 HAMS medium (Sigma N6658). All cultures were supplemented with 1% penicillin/streptomycin (Gibco Life Technologies 15070-063), 10 % (v/v) foetal calf serum (Sigma F7524) and maintained at 37°C, 5 % CO_2_. All reagents were purchased from Sigma unless otherwise stated. Drugs used include rapamycin R8781, 5-fluorouracil (5-FU) F6627 and valproic acid P4543.

### siRNA transfection

Cells were reverse transfected with 20 nM gene specific siRNAs and non-specific scrambled controls (Dharmacon ISG15: L-004235-03-0005, UBE2L6: L-008569-00-0005, Non-targeting siRNA #1: D-001810-01-20), using Lipofectamine 2000 transfection reagent (Life Technologies 11668500), according to manufacturer's instructions.

### Western blotting and antibodies

Total cellular protein extracts were prepared by trypsinization of cells and lysing in modified RIPA buffer (50 mM Tris HCl (pH 7.4), 150 mM NaCl, 0.25 % sodium deoxycholate, 1 % Igepal, 1 mM EDTA, 1x Pefabloc, 1x protease inhibitor cocktail, 1 mM Na_3_VO_4_, 1 mM NaF). All protein samples (30 or 45 μg) were separated on NuPAGE 4–12 % Bis-Tris gels (Invitrogen Life Technologies NP0322) and electrophoretically transferred onto PVDF membrane using the iBlot gel transfer system (Invitrogen IB1001). Membranes were incubated with anti-LC3 (MBL PD014), anti-UBEL26 (Abcam AP2118a) or anti-ISG15 (CST 2743S) antibodies overnight at 4°C and with anti-β-actin (loading control) (Sigma A5441) for one hour at room temperature. Proteins were visualized using relevant IR-Dye conjugated secondary antibodies (Rockland) on the Odyssey IR imaging system (Li-Cor, Cambridge, United Kingdom). For all Western blots, integrated intensities are representative of three independent experiments.

### Cyto-ID autophagy detection kit

Trypsinized cells were stained with the Cyto-ID autophagy detection kit (Enzo Life Sciences ENZ-51031-K200) according to the manufacturer's instructions. An increase in the number of autophagic vesicles is detected as an increase in fluorescence in the 488-2 channel. Data was collected on the BD LSR II flow cytometer with BD FACS Diva acquisition software. Gating and overlay histograms were generated using Flowjo data analysis software.

### Evaluation of morphology

Cells were cytospun and stained using pro-diff I and II (Braidwood Laboratories BAPROD1). Apoptotic cells were characterised based on the appearance of two or more of the following features: cell shrinkage, chromatin condensation, DNA degradation and fragmentation into ‘apoptotic bodies’ within an intact plasma membrane. Cytospin images are representative of at least three independent experiments. Images were captured using a DP70 digital microscope camera and Olympus DP-Soft823 version 3.2 software (Mason Technologies Dublin, Ireland).

### Colony formation assay

Colony formation (clonogenic) assays were used to determine the ability of cells to recover from a drug treatment and re-establish colonies as a monolayer. Cells were transfected or treated and following drug removal, the cells were trypsinized and viable cells counted. 1,500 viable cells were reseeded into a well of a six-well plate (in triplicate). Cells were allowed to attach and grow for between 10 and 14 days. Following the recovery period, media was removed and following a PBS wash, cells were fixed in 96 % ethanol for 5 minutes and stained with Prodiff II (Braidwood Laboratories E310). Plates were scanned and quantified using the Odyssey IR imaging system (Li-Cor, Cambridge, United Kingdom). Results are presented as integrated intensity ± SEM (standard error of the mean) from at least three independent experiments.

### Evaluation of caspase-3 activity by flow cytometry

Following fixation in 4 % PFA, cells were washed in a permeabilisation buffer (0.1 % Triton X-100, 0.1 % sodium azide, 10 mM HEPES, 4 % FCS, 150 mM NaCl) and incubated on ice with anti-active caspase-3 antibody (BD Biosciences 559565) for 1 hr followed by Alexa fluor 488 secondary antibody (Life Technologies A11034). Samples were analysed using the BD LSR II flow cytometer and BD FACS Diva acquisition and analysis software.

### Statistical analysis

Statistical analysis was carried out using GraphPad Prism 5 software. Means were compared using independent student t-tests (unpaired). The p-value was considered statistically significant when it was *p < 0.05, **p < 0.01, ***p < 0.0001.

## SUPPLEMENTARY MATERIALS AND METHODS FIGURES



## Abbreviations

ISG15: Interferon stimulated gene 15
UBE2L6: Ubiquitin/ISG15 conjugating enzyme E2L6
MFI: Mean Fluorescence Intensity
IFN: Interferon
USP18: Ubiquitin-specific peptidase 18
LC3: Microtubule-associated protein 1 light chain 3 alpha.
