# Correction: UBE2L6/UBCH8 and ISG15 attenuate autophagy in esophageal cancer cells

**DOI:** 10.18632/oncotarget.27382

**Published:** 2020-01-14

**Authors:** Chloe M. Falvey, Tracey R. O’Donovan, Shereen El-Mashed, Michelle J. Nyhan, Seamus O’Reilly, Sharon L. McKenna

**Affiliations:** ^1^ Cork Cancer Research Centre, University College Cork, Cork, Ireland; ^2^ Department of Oncology, Cork University Hospital, Cork, Ireland; ^3^ Lecturer in Histopathology, National Liver Institute, Menoufia University, Al Minufya, Egypt

**This article has been corrected:** The affiliation of the 3rd author has been updated. The correct affiliation information is given below:

^3^ Lecturer in Histopathology, National liver institute, Menoufia University, Al Minufya, Egypt

Original article: Oncotarget. 2017; 8:23479–23491. 23479-23491. https://doi.org/10.18632/oncotarget.15182

