# Supplementary material for: UBE2L6/UBCH8 and ISG15 attenuate autophagy in esophageal cancer cells
Source: Oncotarget. 2017 Feb 8;8(14):23479–91. doi: 10.18632/oncotarget.15182 (PMC5410320; doi:10.18632/oncotarget.15182)
Supplement: Supplementary file 1 [file oncotarget-08-23479-s001.pdf]

UBE2L6/UBCH8 and ISG15 attenuate autophagy in esophageal cancer cells

SUPPLEMENTARY MATERIALS AND METHODS

RNA extraction/oligonucleotide microarrays

Affymetrix gene array analysis of each cell line, OE19, OE33, OE21 and KYSE450, was conducted in triplicate (12 arrays – each an independent mRNA extraction). Total RNA was extracted using the RNeasy mini kit (Qiagen, Germany) according to manufacturer's instructions. Purified total RNA was dissolved in RNase-free water and stored at -80°C. RNA sample quality was tabulated by bioanalyser and biophotometric quality control criteria. *In vitro* cDNA synthesis, biotin labeling, transcription and fragmentation were then outsourced to Almac Diagnostics, UK (www.almac.com). Probes were hybridized to the Affymetrix GeneChip® Human Genome U133 Plus 2.0 array. All Eukaryotic Target Preparations were carried out in accordance with the Affymetrix GeneChip® Expression Analysis Technical Manual. These methods are summarised below.

Target preparation one-cycle target labelling assay - cRNA synthesis

2µg of total RNA was converted to cDNA via first and second strand synthesis using the GeneChip® Expression 3'-Amplification One-Cycle cDNA Synthesis kit, in conjunction with the GeneChip® Eukaryotic Poly-A RNA Control Kit. Cleanup of the double-stranded cDNA was carried out using the GeneChip® Sample Cleanup Module. Biotin labeled cRNA was synthesised from the double-stranded cDNA using the GeneChip® Expression 3'-Amplification *In Vitro* Transcription (IVT) Labeling Kit. Unincorporated NTPs were removed with the GeneChip® Sample Cleanup Module. cRNA quality was assessed using an Eppendorf Biophotmeter and an Agilent 2100 bioanalyzer.

Fragmented cRNA generation

25µg of cRNA generated in the IVT reaction was fragmented using 5X Fragmentation buffer and RNase-free water contained within the GeneChip® Sample Cleanup Module. The fragmentation reaction was carried out at 94°C for 35 mins to generate 35-200 base fragments for hybridisation. The fragmented cRNA quality was assessed using an Agilent 2100 bioanalyzer.

Affymetrix hybridisation protocol

Prior to hybridization, the adjusted cRNA yield in the fragmentation reaction was calculated to account for carryover of total RNA in the IVT reaction. 15µg of fragmented cRNA was made into a hybridisation cocktail in accordance with the Affymetrix technical manual corresponding to a 49 format (standard) /64 format array. The hybridisation cocktail was added to the appropriate array and hybridised for 16hrs at 45°C.

Data analysis and generation of gene groups

The filter system used was the flag filter: the starting gene list was all genes. In the first filter, genes displaying a match in terms of their regulation, in OE21 and OE33 cells were retained [Supplementary Figure 1]. Genes that have conflicting regulation i.e. are down regulated in the OE21 cell line but up regulated in the OE33 cell line are discarded. 15805 genes passed this filter, and these comprise the initial 'common to Group A' (CA) gene list. Likewise, a similar analysis of genes in the OE19 and KYSE450 cells revealed a gene list of 16346 common genes, comprising the initial 'common to Group B' (CB) gene list. These two gene lists were further analysed for patterns that were consistently different between CA and CB. This list was filtered, so that only those genes with a differential in expression of three fold or higher, form the final list of ~ 230 genes. In addition, 30 genes were expressed in CA, and not expressed in CB (or vice versa). In total, 260 genes showed greater than a 3 fold difference in expression between the groups.

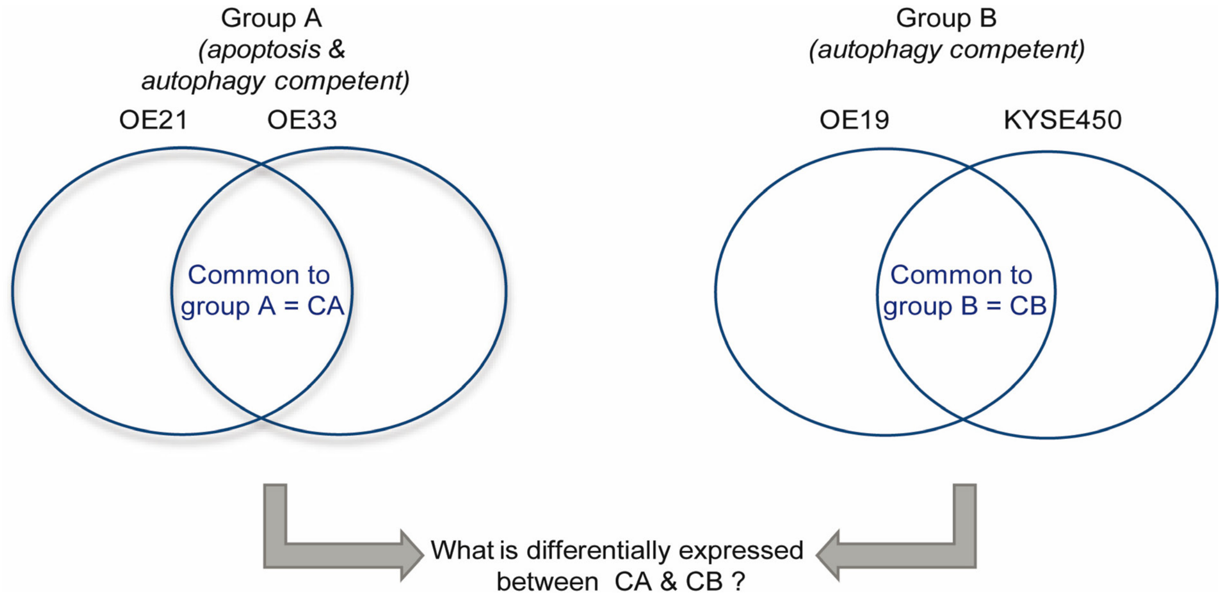

Supplementary Figure 1: Diagrammatic representation of gene array analysis and methods used to generate the 3-fold differential expression gene list.

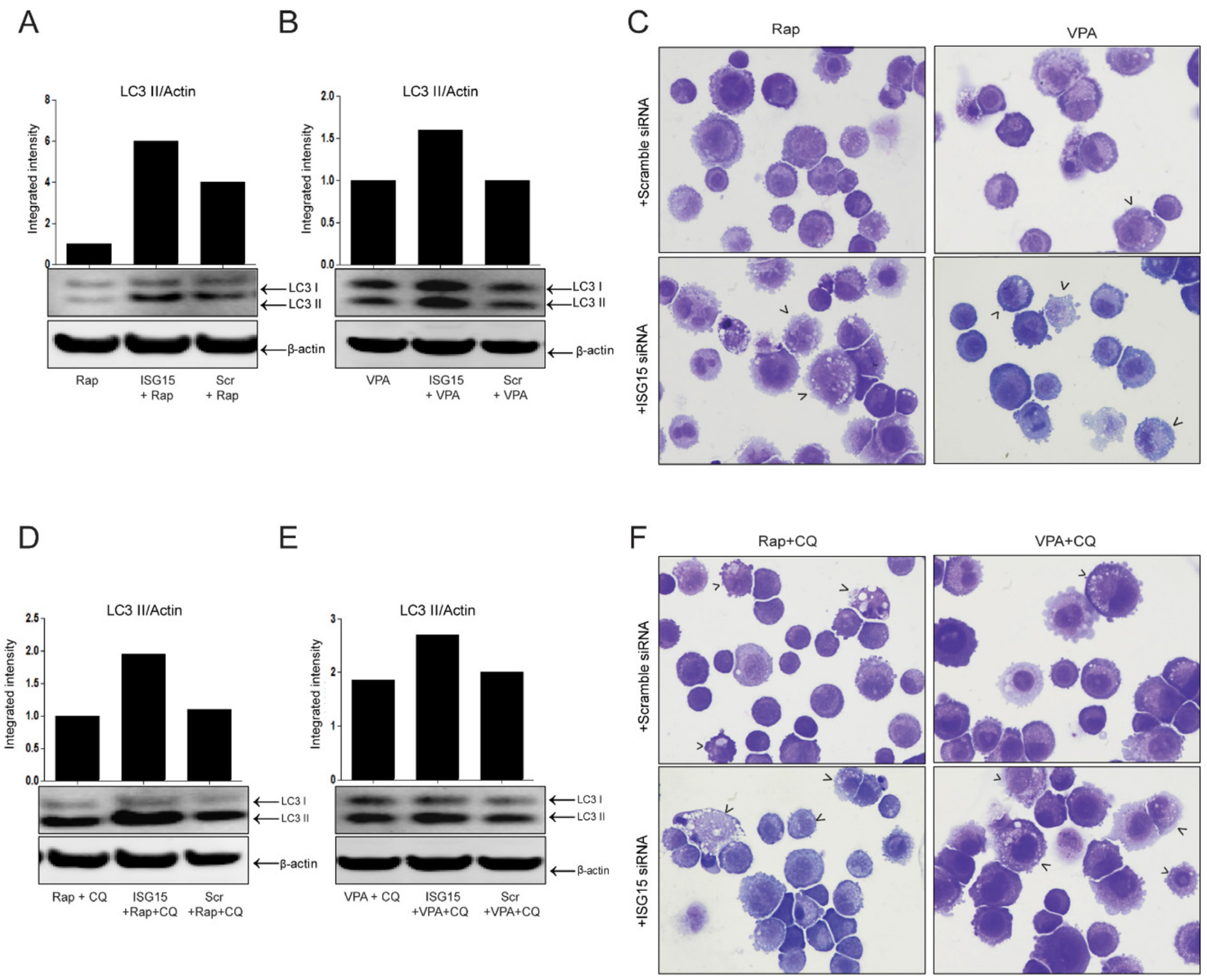

Supplementary Figure 2: ISG15 siRNA enhances rapamycin- and valproic acid (VPA)-induced autophagic flux in OE21 cells. The effect of ISG15 siRNA (middle lanes) on **A.** rapamycin-induced (200 nM) and **B.** VPA-induced (3 mM) LC3 II, relative to untransfected (left lanes) and scrambled control (right lanes) was assessed by Western blot. **C.** Analysis of cellular morphology by light microscopy (40x magnification) compared vesicle accumulation (black arrowheads) in scrambled (upper panels) and ISG15 siRNA (lower panels) cells treated with either rapamycin (left panels) or VPA (right panels). **D.** Autophagic flux was assessed by measuring LC3 II levels in untransfected (left lanes), scrambled control (right lanes) and ISG15 siRNA (middle lanes) cells following 24 hr treatment with chloroquine (10 µM) in combination with rapamycin or **E.** in combination with VPA. LC3 II levels were normalised to β-actin and presented graphically as integrated intensities. **F.** Induction of autophagy was confirmed by morphological analysis (black arrowheads, magnification 40 X) following transfection with scrambled control (upper panels) or ISG15 (lower panels) siRNA in cells treated with chloroquine in combination with rapamycin (left panels) or in combination with VPA (right panels).

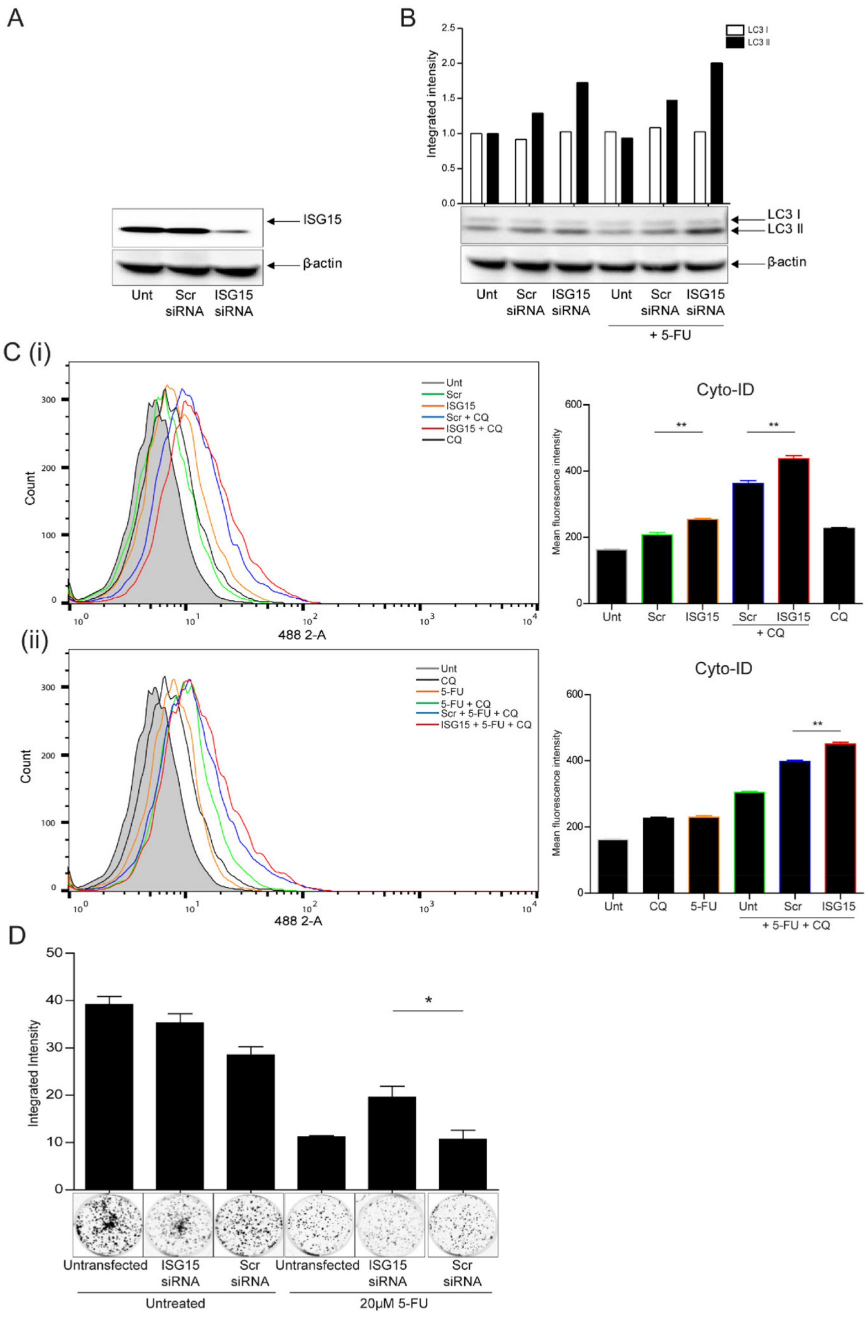

Supplementary Figure 3: ISG15 silencing with siRNA promotes both endogenous and 5-fluorouracil (5-FU)-induced autophagic flux in FLO-1 cells. For all autophagy assays – FLO-1 cells were treated for 48 hr and allowed to recover for an additional 24 hr in drug free medium. **A.** Gene specific siRNA (20 nM) was used to deplete ISG15 expression and silencing was confirmed by Western blot. **B.** The effect of ISG15 siRNA on endogenous LC3 I & II (lane 3) and 5-FU-induced (20 μM) LC3 I & II (lane 6), relative to untransfected (lanes 1 & 4) and scrambled control (lanes 2 & 5) was assessed by Western blot. LC3 I & II bands were quantified, normalised to β-actin and presented as integrated intensities. **C.** (i) Cyto-ID autophagy detection kit was used to assess autophagosome formation by ISG15 siRNA alone (orange overlay) relative to scrambled (green overlay) (**$p < 0.005$) or untreated cells (grey filled histogram). Autophagic flux was assessed by measuring autophagosome formation, following treatment with chloroquine (10 μM) of untransfected (black overlay), scrambled control (blue overlay) and ISG15 siRNA (red overlay) cells (**$p < 0.005$). (ii) Enhanced autophagosome formation was observed in ISG15 depleted cells treated with a combination of 5-FU and chloroquine (red overlay) relative to untransfected (green overlay) and scramble control (blue overlay) cells (**$p < 0.005$). **D.** Following drug removal, a colony formation assay was carried out to determine the ability of ISG15 depleted cells to recover, relative to scramble control cells. Colonies were stained using Rapi-diff and quantified using the Odyssey Infra-red imaging system. Triplicate data is presented as integrated intensity ± SEM (*$p < 0.05$).

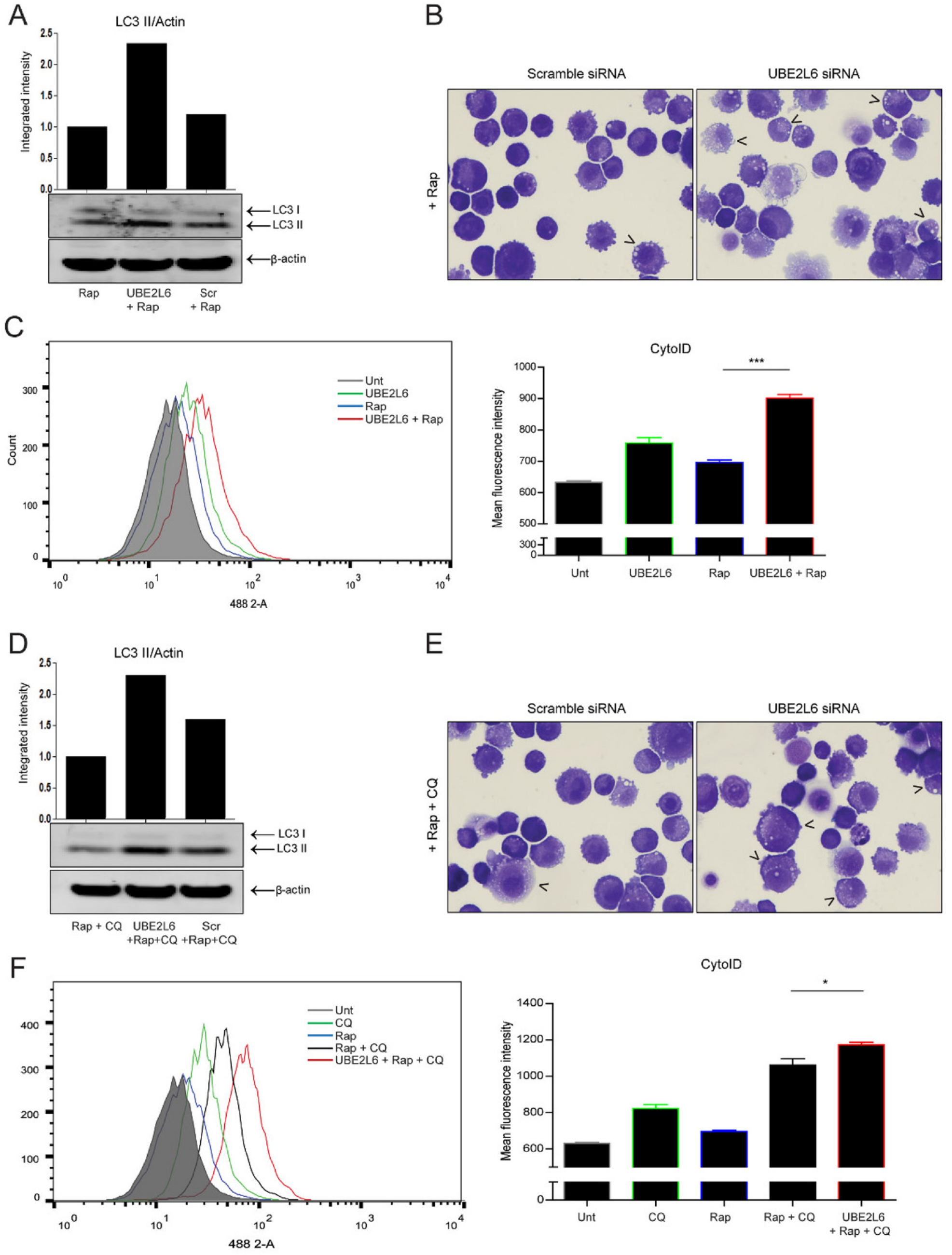

Supplementary Figure 4: UBE2L6 siRNA enhances rapamycin-induced autophagic flux in OE21 cells. A. The effect of UBE2L6 siRNA (middle lane) on autophagy induction relative to untransfected or scrambled controls (left and right lanes respectively) following treatment with rapamycin (200 nM) for 24 hr, and 24 hr recovery was assessed by Western blot. **B.** Morphological analysis (40x magnification) compares vesicle accumulation (black arrowheads) in scrambled siRNA control cells (left panel) to UBE2L6 siRNA cells (right panel) following treatment with rapamycin. **C.** Cells treated with rapamycin (blue overlay) were stained with Cyto-ID. Enhanced autophagosome formation was observed UBE2L6 siRNA cells (green overlay), which was enhanced by the addition of rapamycin (red overlay). Data from three independent experiments is presented as mean fluorescence intensity ± SEM (***$p < 0.0001$). **D.** To assess flux, cells were treated with chloroquine (10 μM) in combination with rapamycin for 24 hr in the presence of either scrambled (lane 3) or UBE2L6 siRNA (lane 2). LC3 II accumulation was normalised to β-actin and data is presented as integrated intensity. **E.** Morphology of cells treated with a combination of rapamycin and chloroquine was compared in scrambled control cells (left panel) and UBE2L6 siRNA cells (right panel). Autophagic vesicles are indicated by black arrowheads (40x magnification). **F.** The effect of UBE2L6 siRNA on autophagic flux was assessed by treating cells with either rapamycin (blue overlay) or chloroquine (green overlay) alone or a combination of both in the absence (black overlay) or presence (red overlay) of UBE2L6 siRNA. Mean Fluorescence Intensities from three independent experiments are presented to the right (*$p = 0.0386$).

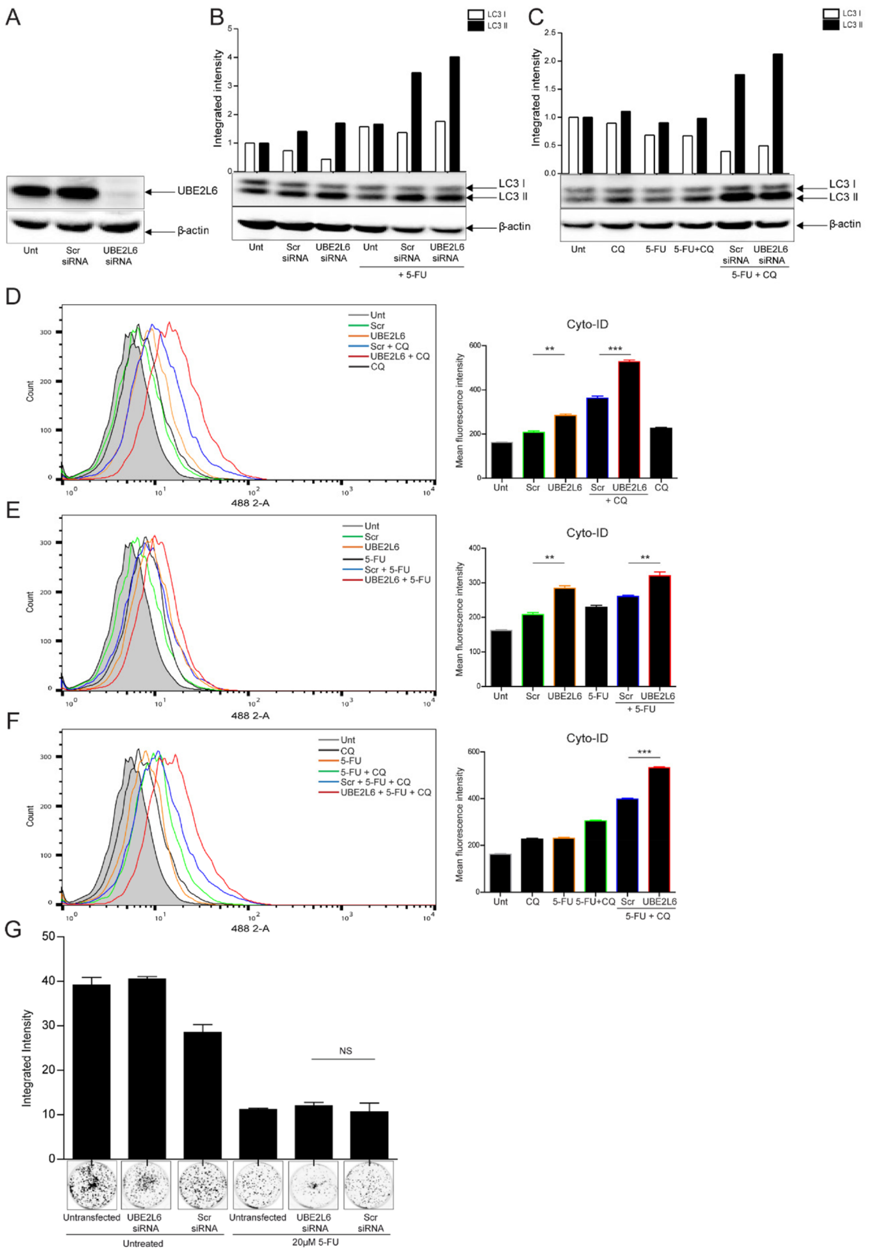

Supplementary Figure 5: UBE2L6 silencing with siRNA promotes both endogenous and 5-fluorouracil (5-FU)-induced autophagic flux in FLO-1 cells. For all autophagy assays FLO-1 cells were treated for 48 hr and allowed to recover for an additional 24 hr in drug free medium. **A.** Gene specific siRNA (20 nM) was used to deplete UBE2L6 expression and silencing was confirmed by Western blot. **B.** UBE2L6 siRNA induced both endogenous LC3 II (lane 3) and 5-FU-induced (20 µM) LC3 II (lane 6), relative to untransfected (lanes 1 & 4) and scrambled control (lanes 2 & 5) as assessed by Western blot. **C.** To assess flux, cells were treated with chloroquine (10 µM) in combination with 5-FU for 48 hr in the presence of either scrambled (lane 5) or UBE2L6 siRNA (lane 6). LC3 I & II bands were quantified, normalised to β-actin and presented as integrated intensity. **D.** Cyto-ID assay was used to confirm autophagy induction by UBE2L6 siRNA alone (orange overlay) relative to scrambled (green overlay) (**$p < 0.005$) or untransfected cells (grey filled histogram). Autophagic flux was assessed by measuring autophagosome formation, following treatment with chloroquine of untransfected (black overlay), scrambled control (blue overlay) and UBE2L6 siRNA (red overlay) cells (***$p < 0.0005$). **E.** Enhanced autophagosome formation was observed in UBE2L6 depleted cells treated with 5-FU (red overlay) relative to untransfected (black overlay) and scramble control (blue overlay) cells (**$p < 0.01$). **F.** Induced autophagic flux was confirmed in UBE2L6 depleted cells treated with a combination of 5-FU and chloroquine (red overlay) relative to untransfected (green overlay) and scramble control (blue overlay) cells (***$p < 0.0001$). **G.** Cells were treated with 5-FU for 48 hr. Following drug removal, a colony formation assay was carried out to determine the ability of UBE2L6 depleted cells to recover, relative to scramble control cells. Colonies were stained using Rapi-diff and quantified using the Odyssey Infra-red imaging system. Triplicate data is presented as integrated intensity ± SEM (*$p < 0.05$).

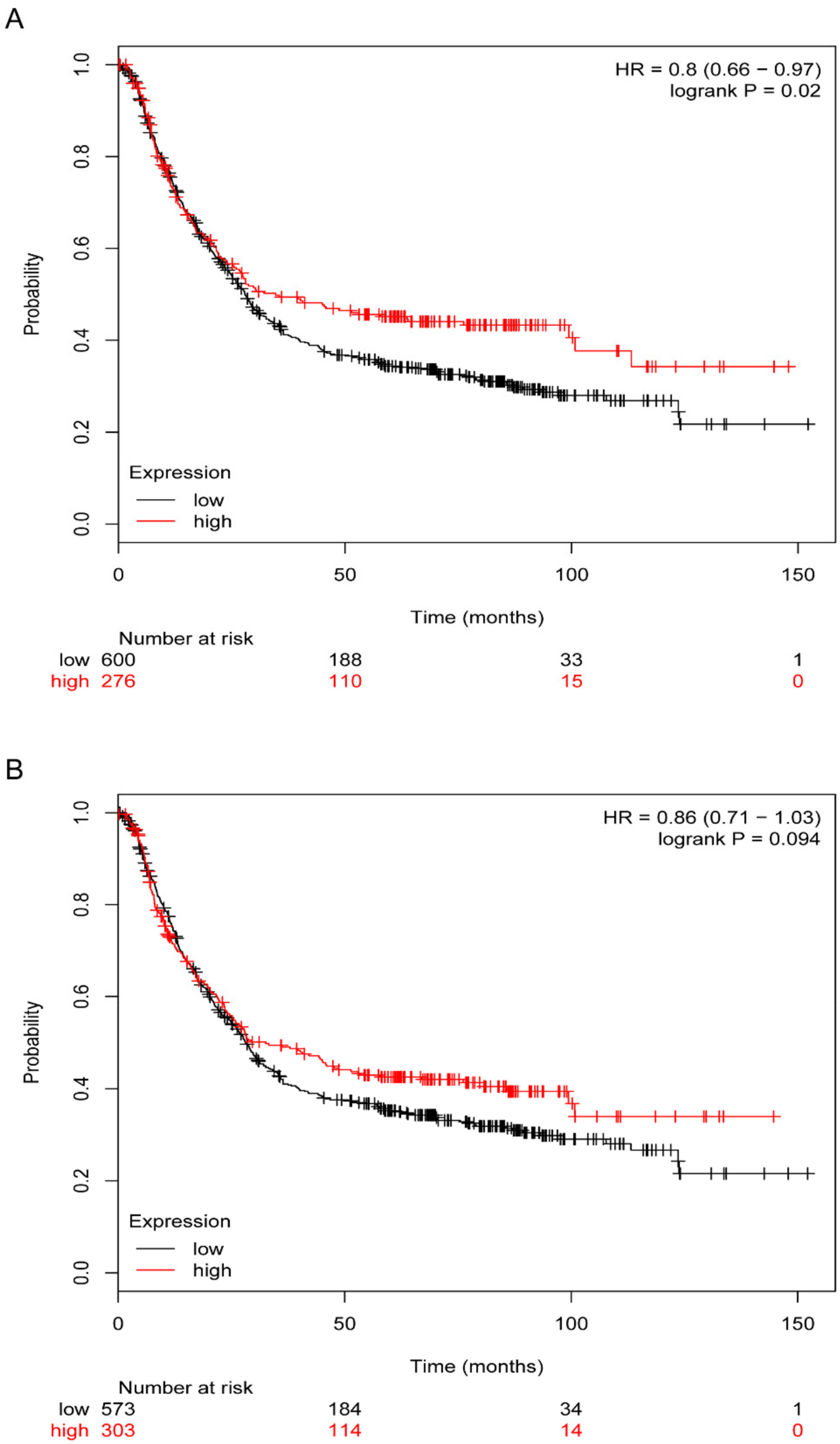

Supplementary Figure 6: Evaluation of mRNA expression in tumours. A. Expression of UBE2L6/UBCH8 (Affy ID201649_at) was analysed in 876 patients from the gastric cancer combined database – assembled from several sources – available at http://kmplot.com/analysis/index.php?p=service&cancer=gastric Kaplan Meier overall survival analysis demonstrates higher expression of UBE2L6 was associated with improved survival (harazd ratio (HR) = 0.8, p = 0.02). **B.** Expression of ISG15 was also analysed in the same patient cohert – and while Kaplan Meier overall survival demonstrates a similar trend between expression and survival – this is not significant (HR = 0.86, p = 0.094).
